# Homicide in Insanity

**Published:** 1858-01-01

**Authors:** John P. Gray


### Art. IX.—HOMICIDE IN INSANITY.

BY JOHN P. GRAY, M.D.

*Read before the Association of Medical Superintendents of American Institutions for the Insane.**

A DISPOSITION to violence is a common characteristic of mental disease. It is exhibited in every conceivable manner, from harsh words to suicide and the most cruel and brutal murders, and is found in every form of insanity. If, then, among the unhappy phenomena or symptoms developed under the influence of the delusions and hallucinations peculiar to the disease, we meet with a tendency so universal, so destructive of happiness, and so dangerous to society, how important is its careful study, with reference to the welfare both of the patient and the public!

If, however, as some assert, neither delusion nor hallucination is necessary to the development of a tendency to violence; that it may burst forth in its most terrible forms suddenly, without premonition, and even without the accompaniment of apparent physical derangement; that the first and only manifestation of disease may be a blind, irresistible desire to take life,—then the subject is of still deeper and more vital importance, and insanity is invested with a degree of interest which pertains to no other disease. If a passing fit of indigestion, or a slight modification in the character or quantity of the blood circulating through the brain, be all that is required to beget suicide and murder, what a lesson on the frailty of man, and yet, at the same time, how impressively does this sad feature of mental disease teach us the dignity and value of reason!

It is of the first importance, therefore, to know whether these serious consequences are *always* preceded by appreciable physical and mental disturbance, or whether they may appear without either; in other words, whether there is ample foundation, in well-attested facts, for the belief that there is such a thing as a sudden explosion of homicidal impulse, independent of appreciable disease—a moral mania, having for its essence a desire to kill.

We propose, therefore, in this paper, to bring before the profession a series of cases treated in the institution at Utica, for the past seven years; not so much, however, with the view of discussing the question of homicide, but rather as a contribution to the general stock of facts already recorded on this important subject. The cases selected are those which have passed under the writer's own observation, and with the details of which he is

* From the "American Journal of Insanity" for October, 1857.

personally familiar. It may be remarked further, that the experience of a State institution so large as that at Utica (having treated nearly five thousand cases of insanity in a period of fourteen years) must be valuable in this connexion—more especially when it is remembered that, by law, all those in the state acquitted of criminal charges by reason of insanity, as well as those under indictment, and found to be insane previous to trial, are sent, together with a large number of dangerous and homicidal cases committed by justices or by friends. Indeed, the cases of homicide or homicidal attempts coming by order of the courts alone, in a population of over three millions of people, must be a valuable record.

For the sake of convenience, the cases are arranged in three classes:

1st. Those who have committed homicide, and who have been placed in the asylum under an order of the Court, or by friends.

2nd. Those who have made homicidal attempts under circumstances which led physicians and others to regard the homicidal tendency as the distinguishing feature of the disease, and some of which were considered as cases of pure homicidal monomania, and as such were committed to the care of the institution by official orders or otherwise.

3rd. Miscellaneous cases, illustrating certain points having a bearing upon the subject under consideration.

I. *Those who have committed homicide.*

Under this classification we have a list of twenty-four patients—nineteen males and five females—whose violence resulted in the death of thirty-three persons.

CASE I.—S., admitted May, 1843. Male, aged 32, labourer, no education, no religious belief, a man of bad habits; form of mental disease, chronic mania following prolonged ill-health; killed the adopted son of his brother-in-law by repeated stabs with a pitchfork and knife. The murder was premeditated, well-arranged plans of concealment were laid and carried out, the instruments were carefully washed, and the body buried. His motives were grounded in personal hatred and revenge. He had always borne the reputation of being a bad man. The act was committed in the daytime. Is still in the asylum.

CASE II.—H., admitted September, 1843. Female, mother of a family, aged 44, of common education, good habits, and even temper. Form of mental disease, paroxysmal mania, commencing at the climacteric period. During one of her paroxysms, while in a furiously maniacal state, she cut the throats of two of her children, and attempted the life of her husband.

When homicidal was always suicidal. The act was committed in the daytime. Still in the asylum.

Case III.—K., admitted May, 1845. Male, aged 40, a cabinet-maker, of intemperate habits. Under the delusion that he was the object of plots and evil designs, he killed a neighbour's wife by stabbing and burning. Act committed in the daytime. Is still in the institution, a demented, dangerous man.

Case IV.—W., admitted August, 1846. Male, aged 25, a boatman, no education or religious belief, but of fair morals, and an industrious man. The hereditary tendencies in the case are not known. In a paroxysm of violence and insanity, killed a man during the daytime on board boat. Was tried and sent to the State prison, where he was found to be insane. Had there frequent paroxysms of insanity, in which he made desperate assaults upon several persons. Is still in the asylum, a very violent and dangerous man. His epileptic paroxysms succeed each other at brief intervals, and mark his periods of violence.

Case V.—K., admitted October, 1848. Male, aged 40, a wealthy land speculator, of intemperate habits; was a violent, revengeful, suspicious man. At the first outbreak of an attack of acute mania, stabbed a man in the street, in the daytime, under the delusion that he was following his footsteps to rob him. Recovered, and eloped from the asylum in 1852.

Case VI.—B., admitted January, 1850. Male, aged 50, farmer, of violent and ungoverned temper, a drunkard; was labouring under chronic mania, caused by his prolonged intemperance. One morning openly stabbed a neighbour, under the delusion that he was unkindly disposed to him. Was tried, convicted, and sentenced to prison for life. Was there found to be insane, and sent to the asylum, whence, after three years' confinement, he escaped, and died on his way home. Was a very dangerous man, constantly secreting and making instruments with which to kill those whom he disliked.

Case VII.—F., admitted October, 1850. Male, aged 30, a labourer, of good habits, of hereditary taint. Had an attack of acute mania, induced by fatigue and anxiety; the first indication of which was a maniacal frenzy, during which, as a matter of resistance, under the delusion that he was about to be murdered, he stabbed two men on the deck of a steamboat. Act was committed in the daytime. Patient recovered, and was discharged.

Case VIII.—H., Sandwich Islander, admitted November, 1850. Male, aged 30, a sailor, of good habits, educated. His purse was stolen by the steward of the vessel while in port; and under the impression that he was doing right, he stabbed the steward, when an attempt was made to arrest him. In maniacal frenzy he killed two men by stabbing, and wounded many others; was finally shot down and captured. Acute mania followed. Was acquitted on the ground of insanity. Recovered and was discharged.

CASE IX.—P., admitted June, 1851. Male, aged 51, of intemperate habits; had an attack of melancholia, induced by his vicious indulgences. One morning after breakfast, shot a neighbour, under the delusion that he was plotting against him. Gave himself up to the authorities, evincing no regret or sorrow. Recovered, and was discharged.

CASE X.—N., admitted March, 1852. Female, aged 58, of insane parentage, a worthy member of the Baptist church. Had an attack of sub-acute mania, caused by domestic trouble. In a paroxysm of maniacal passion she killed a neighbour's child by dashing its head against the wall. She was also suicidal. Recovered, and was discharged from Asylum.

CASE XI.—B., admitted April, 1852. Male, aged 32, a gardener, of good habits, inherited insanity from his father. Had an attack of acute mania, caused by domestic trouble, and killed his wife by stabbing, under the delusion that she was not his wife, and was plotting against him. Still in the Asylum, a very dangerous man.

CASE XII.—D., admitted April, 1852. Male, aged 35, a labourer, was an intemperate man, and of intemperate ancestry. Was for some time depressed and suspicious. One morning he went forth deliberately and knocked down and stamped upon a kinsman of his, until he was dead, under the delusion that he was inimical to him. The following day he was seized with acute mania, demented rapidly, and died of general paralysis before the end of the year.

CASE XIII.—D., admitted March, 1853. Male, aged 29, a boatman, of intemperate habits, and of insane parentage; had been in a melancholy, half-demented state for some months. His father went to his room one evening to ascertain whether his son was at home. Having no light, he repeated his name several times. Patient, who was dozing upon his bed, sprang up, thinking his father was shouting for help, seized a club, and encountering him in the dark, killed him by a single blow. Recovered, and was discharged.

CASE XIV.—W., admitted June, 1853. Male, aged 42, a pedlar, of intemperate habits; was demented from long-continued dissipation. He loaded a gun one morning, and under the delusion that he was obeying a command of God, shot a man who was ploughing in a neighbouring field. Was very suicidal, died in the Asylum.

CASE XV.—T., admitted August, 1853. Male, aged 33, a cabinet-maker, of good habits, a Swedenborgian; killed his brother-in-law with an axe. The act was committed in the evening, and in connexion with some family quarrel. On second trial, after prolonged imprisonment, was acquitted on the ground of insanity. Was subsequently discharged by the court as not insane.

CASE XVI.—W., admitted March, 1854. Male, aged 42, farmer, sober and industrious, of a kind and amiable disposition. Hereditary taint in family. Had twice suffered from mental disease. Suddenly, and without assigned cause, became gloomy and depressed; talked much about his soul, and the earthly and future welfare of his family. Read his bible a great deal, and finally secluded himself; and on one occasion prayed for thirty hours in succession, without rising from his knees. Thought he could look directly into heaven, and converse with the Saviour. Suddenly became composed, took his razor very deliberately, and cut his wife's throat to the vertebræ, producing instant death. He then made a similar attack upon his daughter, who, however, escaped from him with the remainder of the family. Patient at once sank into profound stupor, refused food, rapidly emaciated, and was brought to the Asylum demented. Subsequently recovered, retaining only a dreamy recollection of the homicide. Was discharged.

CASE XVII.—S., admitted May, 1854. Male, aged 56, a quiet, industrious man, the father of ten children. Was slightly intemperate, rather reserved in disposition, but kind to his family. Began to complain of intense headache, became jealous of his aged wife, and cross to his children. One morning walked out to the woodpile, procured an axe, returned to the house, knocked down his wife, dragged her to the door, and deliberately cut off her head. The children fled and aroused the neighbours. Patient gave himself up and desired to be hung. On opening of the court, was so evidently insane that he was ordered to the Asylum without trial. When admitted was labouring under dementia. Has recovered; seldom speaks of the act, of which, however, he has a perfect recollection.

CASE XVIII.—A., admitted May, 1854. Male, aged 40, shiftless and uneducated, abjectly poor, lived with and was supported by his sisters. Was addicted to the free use of intoxicating drinks; was of insane parentage. Became silent, pale, and emaciated; soon imagined that he was possessed of great wealth, which his neighbours were trying to get away from him, and under this delusion procured a gun and shot one of them. Was brought to the Asylum, and in a few months died of general paralysis.

CASE XIX.—G., admitted August, 1854. Female, aged 36, of an even temper, and gentle disposition. While nursing child, and in rather delicate health, contracted an ungrounded jealousy of her husband. This continued for two years. At times she was also suspicious of and violent towards others. One morning locked herself in the house and barricaded the windows and doors. Attempts were made to gain admission, when, in a paroxysm of maniacal passion, she seized her children by the feet and dashed their heads against the wall, fracturing the skulls of two, of whom one died. She was brought to the institution in a state of dementia, and was subsequently removed to the country house, unimproved.

Case XX.—T., admitted February, 1855. Male, aged 45, a clergyman, of academic education; of insane parentage, of intemperate habits, and violent, ungovernable disposition. Had an attack of *mania a potu*, during which he made a murderous assault upon his family with a razor. He killed one child, and wounded others. After a trial, in which the jury could not agree, and pending a second, was sent to the Asylum on order of judge. Feigned dementia. He eloped in 1855.

Case XXI.—L., admitted May, 1855. A labourer, of temperate habits, but of violent, passionate disposition. Became very angry with his wife on account of her refusing to sign a deed of conveyance. Subsequently killed her and three children. Committed the act in the daytime. Does not deny it, but says he has his reasons for it. Is demented. Was long imprisoned before being sent to the Asylum. Has a large scrofulous tumour upon his neck. Says that he has always had it. Is still in the Asylum.

Case XXII.—E., admitted October, 1855. Female, aged 35, German, religiously educated, and of gentle disposition; sank into dementia after childbirth. Had a delusion that her husband was not really married to her—that he was an adulterer. On his returning from his work at noon, one day, he lay down to rest while his dinner was preparing. Falling into a light sleep, the wife seized the opportunity to cut off his head with a hoe. Is still in the Asylum, demented.

Case XXIII.—W., admitted March, 1856. Female, aged 27, of insane parentage, religiously educated, member of Episcopal church, of great evenness and gentleness of disposition; was subject to periods of depression, owing to the intemperance of her husband, poverty, disappointment, and home-sickness. Had attempted suicide. One Sabbath morning, and while she was labouring under depression, her husband left to go fishing. In his absence she seized an axe, killed four of her children, and cut her own throat. Is in the Asylum, demented.

Case XXIV.—L., admitted April, 1857. Male, aged 22, a shoemaker, unmarried, of vicious habits. Procured a pistol, went to a road which farmers, returning from market, were accustomed to travel; was invited by a man, whom he supposed to be a farmer with money, to ride with him; rode some distance, then got behind him and shot and robbed him. On preliminary trial was ordered to Asylum, where he remains in a state of dementia.

II. *Those who have made homicidal attempts under circumstances which led physicians and others, in many instances, to regard this homicidal tendency as the distinguishing feature of the disease, and some of which were considered cases of pure homicidal monomania, and as such committed to the institution by official order or otherwise.*

Under this classification twenty-five cases are presented, twenty of which are males, and five females.

CASE I.—S., admitted March, 1846. Male, aged 25, uneducated and vicious. Had epilepsy induced by intemperance. Became quarrelsome, considered himself injured, and in a rage would attempt to stab those whom he disliked. At time of admission to the Asylum, exhibited no marked mental aberration. Soon began to dement; was always a dangerous man; invariably makes his attacks in the daytime, and on persons whom he dislikes, and never threatens or uses violent language. The homicidal tendency is not constant, and is at times attended with strong suicidal disposition, and occurs independently, so far as can be observed, of his epileptic seizures.

CASE II.—R., admitted August, 1847. Male, aged 42, married, the father of five children, common education, not religious, of good moral habits, and an industrious man. Hereditary tendencies not known. For many years had paroxysms of violence, in which he would threaten his family and neighbours; but, upon recovery, was a kind and peaceable man. In one of these periods went to the office of two physicians and attacked them, as he afterwards said, with the intention of killing them, under the impression that they had given him medicines which had injured him. They resisted him, barely escaping with their lives. He was tried, and sentenced to the state prison for the term of six years. Remained for that time in prison, where his attacks of maniacal excitement were frequent, and during which he was violent, attacking those about him suddenly and furiously.

After his return home these paroxysms continued, with the attempts to kill. His physician then detected symptoms of epilepsy, and, on inquiry, ascertained that he had received a blow on the head some years previous, which had left a considerable depression in the occipital region of the skull. He had "frequently thought of operating upon him, thinking it possible that from the blow some portion of the skull might perhaps be pressing upon the brain."

He was committed to Asylum upon an order of the Superintendent of the Poor, and for two years was subject to paroxysms of violence, lasting a few hours, in which he frequently attacked those about him. These paroxysms were usually followed by epileptiform seizures. At other times he was uniformly kind and industrious, and having, during his periods of violence, on several occasions injured patients and attendants, he came at length to anticipate his seizures, and request to be placed in his room, and to have no one approach him suddenly. He experienced at these times pain in the head, a sensation of ringing in his ears, dimness of vision, with a vague idea of impending danger. His violence was occasioned by the delusion that persons were attacking him with the intention of killing him; and on several occasions, while alone in his room, had a distinct sensation of a blow upon the head, when he would immediately begin a furious contest with his imaginary enemies. At the end of six years, during which the violence of his epileptic periods gradually abated, he returned home in a state of partial dementia.

Case III.—L., admitted December, 1847. Patient was an educated man, of gentle and amiable disposition. When he was about thirty-six years old, became changed in character; neglected his work, wandered about, was depressed, and afterwards morose and irritable. One morning walked out of the house, returned with an axe, and made a murderous attack upon his parents. Was taken to prison, and soon after brought to the Asylum. Was a dangerous man, and for a long while retained the most revengeful and deadly hatred of his parents. Subsequently became profoundly demented, and is now a harmless, inoffensive man.

Case IV.—V., admitted July, 1849. Male, aged 25, son of a farmer in good circumstances, and an ordinarily intelligent boy. After scarlet fever health was impaired, and at the age of 20 began to be passionate, and to entertain suspicions of friends and neighbours. Continued to grow worse, though worked regularly on farm. One day took a gun and shot at a neighbour, with the intention of killing him. Was arrested, but considered insane, and sent to the Asylum by order of the court. When received was in a state of dementia, which increased gradually, and he was removed, after a period of three years, a quiet, harmless man.

Case V.—M., male, aged 25, admitted August, 1849. Native of Ireland, no education, was a boy of violent temper, and early placed in the army by wish of his friends. While there, and without known provocation, stabbed a sentinel with his bayonet, and then made his escape, and his friends sent him to this country. He entered the army in this country, and was frequently refractory, requiring discipline. On one occasion he ran his bayonet into a fellow-soldier, wounding him severely but not fatally. He was brought before the proper officers, and discharged from service on the ground of imbecility and insanity. He wandered off, and was placed in an alms-house, where he was very violent and abusive. He was sent to the Asylum on an order of a superintendent of the poor, and was, when received, labouring under dementia. His dangerous propensities continued for some time, but under the progress of his disease he became so inoffensive as to be removed again to the poor-house after two years, where he still remains.

CASE VI.—M., admitted September, 1849. Male, aged 30, married, a lawyer, liberal education, religious, and of irreproachable character. At the age of 25 had an epileptic fit, which was followed by others at somewhat irregular intervals; not, however, impairing his mind perceptibly, though depressing his spirits, and rendering him at times irritable. Two weeks before admission, after several fits, became excited and boisterous, and attempted to kill his mother and sister. They fled and closed a door against him, when he spent his fury upon the glass and furniture. He soon became calm, appreciated his condition, and was willing to be confined in an asylum. Remained under treatment for a time, and returned home, but the recurrence of a violent disposition towards his family induced him to request his sequestration. Subsequently he became demented, and died at the close of a series of epileptic convulsions.

While in the Asylum, was once in the medical office, executing a receipt for rent paid by a tenant, when he suddenly seized a weapon, sprang across the table, and attacked the physician, under the hallucination that he was a man who had once defrauded him, and was present to induce him to yield up some claim to property. He subsequently stated that he seemed to see the man distinctly, and that he heard him speak, and make the request. The physician had not spoken.

CASE VII.—C., admitted March, 1851. Was an uneducated, vicious, intemperate man, of decided hereditary taint. One day, while disputing with his father about some trifling matter, endeavoured to kill him with a spade. Jury found him guilty of an assault with intent to kill, but subsequently he was sent to Asylum. Was labouring under dementia, and died in the institution in 1857.

Case VIII.—T., admitted April, 1851. Wife of labourer, second husband, member of church, good habits, common education, mother of three children. After birth of last child general health impaired and mind somewhat disturbed. Partially recovered; nursed her child sixteen months; was passionately attached to it; attempted to wean it, and in a few days was seized with acute mania; became violent toward husband, and abusive of her child. Tried to drown her second child; subsequently stripped it and put it under the hot stove, and took the eldest into the garden and attempted to smother it by pressing its face into the ground. Was furious on the way to Asylum, and attempted to jump from steamboat into the lake. Recovered, and was discharged in 1852. Retained a dreamy recollection of her conduct.

Case IX.—H., admitted November, 1852. An educated woman, and mother of a large family. Was of an amiable and gentle disposition, but sank into melancholia at the climacteric period. There a strong hereditary taint in her family. One night she requested to sleep at the front of the bed, which was permitted. On retiring, she drew a small stand to the bedside, and when she supposed her husband asleep, cautiously took a razor, which she had concealed in a drawer of the stand, and drew it across his throat. He, however, had not been asleep, and resisted. She then cut her own throat. She never spoke afterwards, but continued very suicidal to the day of her death, which occurred about six months afterwards.

Case X.—M., admitted June, 1852. Male, aged 30, a labourer, of intemperate parentage. Was a bad man and a drunkard, but generally provided well for his family, and lived peaceably with them. Had complained for six or eight months of general indisposition; was subject to gastric disturbance; was depressed in spirits and wakeful at night. Two months before admission, without any apparent motive, he gave his wife a large dose of opium. Observing its effect upon her, he ran to a neighbour, told him that he had poisoned his wife, and insisted upon being killed. On his neighbour's refusing to comply with this request, he seized a razor and cut his throat. His wife was restored. He was brought to the Asylum, gradually sank into profound dementia, and died. Always spoke of his family with much affection and interest.

Case XI.—D., admitted September, 1852. Female, aged 52, wife of a farmer, common education, religious, good habits, hereditary, mother insane for many years, and other members of maternal branch. Late in life married a widower with several grown-up children. Had occasional domestic troubles. At climacteric period became depressed in spirits, with impaired general health; sank into a state of melancholy. Thought her husband desired to get rid of her. Secluded herself much of the time, and frequently laid awake during the night. One morning followed her husband into the wood-house, seized an axe, struck him on the head and felled him. Subsequently inflicted numerous severe blows about his head and face, cutting him horribly. She then gave herself up and requested to be punished. Said she had done an act against the law, but had done no wrong; that her husband was in league with the devil, and had sold her to him; that on several occasions in the night she had observed them talking together, and that the night preceding the morning of the deed, the devil had made a large fire in the centre of the room and kept it burning a long time, waiting for her to go to sleep; that during this time her husband pretended to be asleep, but at the same time she heard his spirit talking and laughing with the devil and arranging her destruction.

This woman rapidly demented, but retained her delusions. She was subsequently removed by her family, and become profoundly demented.

CASE XII.—S., a male, aged 25, a farmer's son, was admitted to the Asylum in December, 1852. There was no inherited disposition to insanity. At the age of eighteen had an attack of mania, from which it appears he recovered but partially, yet went to school for two years, when he had a recurrence of disease. Was subsequently subject to paroxysms of mania, to the time of admission above mentioned. During one of his paroxysms he attacked his father with an axe, and the same day set fire to his father's barn. On admission, was labouring under dementia—was silent, easily controlled, never spoke of his family, when his father or mother visited him usually answered questions, but took no interest. Never would disclose the reasons for his violent conduct. Worked steadily, and gradually became more and more demented, and died in 1857.

CASE XIII.—G., admitted April, 1853. Male, aged 33; a man of good habits; father was insane. Began to talk of his troubles, and accused his brother and sister-in-law, with whom he lived, of being their cause. Under this delusion made a murderous assault upon them with an axe. Was brought to the institution slightly demented, and after a short time was removed; but his feelings towards his brother and sister-in-law at once returned, and he was brought back. As his disease progressed, the homicidal tendency disappeared.

CASE XIV.—F., admitted September, 1853. Male, aged 49; was an eccentric, morose man, and occupied, entirely alone, an isolated farm-house. Began first to shoot at every one who passed his house. Blocked up the road, which happened to be an unfrequented one. Thought that mankind wished to deprive him of his little farm. He wounded no one fatally. Is in the Asylum, and retains the same delusions. Is quite demented.

CASE XV.—G., admitted November, 1853. Male, aged 27, German, member of Lutheran Church, of good education, a musician by profession; was married to a lady of great personal beauty, his superior physically, and to whom he had been long and tenderly attached. Some months after marriage he made an attempt to push her into the canal, and also the river. After several attempts of this kind, she demanded his reasons for such strange conduct. He burst at once into a paroxysm of weeping, mingled with the fondest expressions of endearment, and an obscure reference to the bliss of heaven. She concluded that he was becoming insane, and that, under some delusion, he desired to kill her, and afterwards take his own life. Wishing to avoid the shame and despair of such an exposure, she courageously determined to keep the secret, and rely upon her own strength and presence of mind to prevent the accomplishment of his purpose. He was paler than usual, and suffered from headache, but was able to discharge his accustomed duties. He continued his attempts, his wife searching him every night, often finding a brace of pistols, a razor, or a carving-knife, then locking the door and securing the key. It occurred to her that travelling might benefit him, and they accordingly started to visit some friends at the West. On board the steamboat, crossing Lake Erie, he was most persistent in his efforts to induce her to walk with him on the upper deck, and did not cease begging to have her do so until midnight, and then cried himself to sleep. Having nothing to do, his attempts only increased in frequency. They retired one night, after a most careful search, as usual; when about half asleep, she was aroused by feeling the edge of a razor drawn across her throat. By combining great presence of mind with all the strength she could summon, she escaped with an extensive but fortunately superficial wound; and, to use her own language, "thinking it about time," she brought him to the Asylum. There, one of his first acts was to conceal a razor. His disease was incomplete dementia. He soon recovered, and subsequently acknowledged that his sole and engrossing aim was to kill his wife, and then himself, to secure the mutual enjoyment of heavenly bliss; thinking, as he expressed it, with eyes dancing with delight, "if we were so happy, happy here, what would it be in heaven!" His object in concealing the razor was to cut his wife's throat the first time she should be permitted to visit him, and then his own.

CASE XVI.—N., admitted November, 1853. A clergyman, aged 29, of studious habits. From long-continued mental application, as well as from confinement to an ill-selected and scanty diet, a freak of his eccentric mental constitution, began to experience a gradual impairment of his physical health, and to entertain delusions of the nature of suspicions relating to his clerical duties and his brethren. Attended a meeting of the synod, and on his return his friends noticed him to be labouring under considerable mental disturbance. Declared that on account of his youth he was the object of distrust. Suspected his neighbours desired the separation of his wife from himself, and that they were endeavouring to poison his family. During his resdence in the Asylum the delusions connected with those with whom he was associated led to frequent outbursts of violence and rage. Health gradually improved, and his mental condition so altered that his wife and friends removed him. Soon manifested evidence of a return of his mental disease. Began to threaten his wife and family, and they left him through fear, and he remained with his mother. Without assigning a motive, he procured a knife and attempted to kill her. Was arrested, and after a brief confinement in jail was again removed to Asylum at Utica, where he remains, his condition unimproved.

CASE XVII.—T., admitted December, 1853. A young man, aged 25, of good habits and gentle disposition; lived with his widowed mother, to whom he was tenderly attached. He had at one time an attack of mental disease, had recovered, and remained well for many years. He seemed a little restless; left home, and after several days' absence returned with a gun, which he deliberately loaded, and attempted to discharge at his mother. The cap missed, at which he became so enraged that he broke the gun into fragments. Was secured and placed in jail. His conduct was variable; he was at times noisy and excitable, after which he would become quiet; was filthy in his habits, and refused to walk, talk, or eat. Made several attacks upon his attendants. While under observation in the Asylum he continued much in the same condition as is described above. After a residence of two years, during which time he had not been known to converse or express any wish, requested permission to write to his mother. The letter which was written was both appropriate and affectionate. From this time his improvement was gradual, and he was discharged after a residence of three years. The delusions this patient had so long entertained were never unfolded.

CASE XVIII.—B., admitted June, 1854. A merchant, aged 68, married, of good education, but of intemperate habits. Began to manifest evidences of insanity six years prior to admission to the Asylum. Hereditary predisposition to mental disease existed in the paternal branch of the family. The early indications of the disease were found in his restlessness, and peevishness of manner; disposition to engage in law-suits; mismanagement of his business, resulting in its derangement and in great pecuniary losses; and in his suspicions of those about him. Under the delusion that he was about to be robbed, he procured arms, and on slight provocation discharged his pistols, which he at first loaded with powder, then with paper balls, and eventually with shot, at passers-by. On being arrested and searched, two revolvers, heavily charged, were found upon his person. Had always enjoyed an excellent reputation, but had gradually become passionate, and acquired exalted ideas of property. When it was first proposed to him to go to an asylum, he declared he would commit suicide, but afterwards went without difficulty and willingly. After admission became gradually quiet and easily controlled, and, after four months' residence, returned to his family.

Case XIX.—C., admitted September, 1854. A farmer, aged 56, of limited education, intemperate in his habits, as well as eccentric and passionate. Gave indications of insanity two years prior to admission. On several occasions attacked his neighbours without provocation. Steps were taken to place him in an asylum, but he made his escape. Returned after an interval, and continued quiet till about one week before his reception, when he attacked a neighbour with a club, injuring him severely. Subsequently attempted to shoot his wife. She escaped by running away. He laboured under the suspicion that plots existed against his life and property. Since his admission into the Asylum has had an apoplectic seizure. He rarely speaks to any one, but is disposed to seclude himself, and devotes the greater portion of his time to reading. He is regarded as a dangerous man.

Case XX.—L., admitted October, 1855. A farmer, who had been known as an intemperate, violent, and dangerous man for fifteen years, during the greater portion of which time he had supported himself and accumulated a small property by his industry. Began to manifest indications of insanity about four months prior to admission. His violent conduct was such as to excite the apprehension of persons having occasion to pass his house, as well as his neighbours, whom he was in the habit of threatening with violence. On several occasions fired at persons who passed near his house, under the delusion that he was to be attacked and robbed of his money, and an invention for exhibiting a perpetual motion, which he supposed he had perfected. An officer, with the assistance of a number of persons, went to the house for the purpose of arresting him. It had been barricaded, and a defence prepared with loaded pistols and guns, a handspike, clubs, a basket of stones, an axe, and a large pot of boiling water. He yielded after a desperate resistance. Was confined for nine months, gradually overcoming his delusions, when his friends and neighbours removed him.

CASE XXI.—W., admitted August, 1855. Aged 42, mother of several children, of liberal education, member of church, good habits, strong hereditary predisposition to insanity in family. After birth of last child was irritable, at times maniacal, entertained unfounded suspicions of her husband, neglected her domestic duties, secluded herself. Secreted a carving knife, and in the night attempted the life of her husband. Labouring under chronic mania. Is still in Asylum.

CASE XXII.—W., admitted December, 1855. A carpenter, aged 35, of intemperate habits and bad morals. Gave evidences of insanity nine months previous to this time. Insanity hereditary on paternal side. Became violently insane; fired a pistol at a young man in the street, and subsequently shot at two men riding in a buggy, and made other homicidal attempts. When arrested had a heavily loaded pistol, a hand-saw, and a steel square in his bed with him, with which he made a desperate resistance. He entertained delusions concerning his property and the chastity of his wife. Returned to his family in July, 1856.

CASE XXIII.—W., admitted June, 1856. Aged 42, wife of a captain of one of the Hudson River boats. She was a lady of intelligence, much respected for her amiability and virtues, and for many years a worthy and consistent member of the Baptist church. She was of healthy parentage, though one paternal uncle and two aunts had been insane. Her domestic relations had always been very pleasant.

She resided with her family during the summer of 1855, in a malarial district. Her second child was seized with intermittent fever, and died in August; her eldest son, to whom she was passionately attached, also suffered severely from the disease, and in the following month had a series of convulsions which left him amaurotic. The entire winter was spent in the most devoted and assiduous attention to this child, under the hope, held out by his physician, that his sight would return to him when he had recovered his usual health. After months of care and watchful nursing he eventually regained his previous flesh and strength, but was still unable to see.

On the opening of navigation, she left home with him to submit his case to the oculists of the city of New York, confidently believing that their great experience and superior skill would restore his sight. They, however, pronounced him hopelessly blind. She returned home overwhelmed with grief, feared that she had loved her children too fondly, and in her engrossing affection for them had neglected her duty to her God, who, to punish her idolatry, had taken one away from her, and smitten the other with blindness. In her sorrow she sought the advice and counsel of her pastor, presented to him her view of the affliction, and suggested fasting and prayer, to which she understood him to assent. She accordingly fasted for three days, spending most of the time in meditation in her own room, in reading the Scriptures, and prayer. She was more cheerful on the evening of the third day, but passed a restless night, and on the following morning was more uneasy, felt feverish, and, in the opinion of a neighbour, who called upon her, seemed to talk more than usual, and in a hurried, excited manner. During the remainder of the week she was as cheerful and composed as she had been at any time for some weeks previously, though feeling faint from her prolonged abstinence. On the morning of the following Sabbath she attended church and listened to a sermon, the theme of which was the sacrifice of Isaac by Abraham, and returned home greatly excited. At evening service, as was occasionally the custom for females in her church, she arose, spoke of her afflictions, the light in which she regarded them, &c. Becoming somewhat excited, and weeping violently and hysterically, she was advised to return home, and did so, with one of her friends, her husband at this time being absent in command of his vessel. She could not distinctly recall the state of her feelings, nor how she rested during this night. On the following morning she was detected approaching her children's bed with a large carving-knife in her hand. She was greatly agitated, but at once acknowledged her intention of sacrificing her children, in order to show her submission to Divine will, as Abraham had. Her friends explained to her the manifest insanity of such an idea, and she came to the institution voluntarily. She was pale and nervous, trembling whenever addressed; but under the use of tonics and laxatives rapidly recovered, and soon returned to her family.

Case XXIV.—W., admitted August, 1856. Male, aged 35, of temperate habits, and a professor of religion. Having purchased a farm, found himself embarrassed in meeting his pecuniary obligations, and applied himself very closely to work. About two months previous to his admission his wife noticed his actions to be unusual, and that he worked and slept irregularly. In this state he undertook the care of a sick person, which impaired his physical strength. Two weeks prior to admission he took one of his children and a dog, and proceeded to the woods, where he hung and quartered the dog. Intended to proceed in the same manner with the child, but, under the impression that he had brought the wrong one with him, returned to his house for another, deliberately telling what he had done and intended to do. His wife secured the children, and he was placed in security till his removal to the Asylum could be effected. The delusion under which he laboured was, that he was directed by God to exhibit a proof of his religious devotion. He remained under treatment seven months, and was discharged in his usual health.

CASE XXV.—S., admitted April, 1857. Male, aged 37, farmer, member of the Methodist church, industrious in habits, and of good morals. Showed indications of mental disease one year prior to admission. There exists hereditary predisposition. Nine months prior to reception began to neglect his farm and family. The greater portion of the time sat in the house and read the Bible, seldom conversing with any one, and objected to his food for days together. Burned candles during three successive days and nights. At about two o'clock one morning he left his wife asleep, as he supposed, and went to his barn, killed a heifer, and drew it into a field. He then returned to his wife's chamber and, after satisfying himself that she and the children were asleep, fastened the doors and left the house. From the house he went to the barn, and slaughtered a number of sheep, and again returned to the house, with an axe, with the intention, afterwards avowed, of killing his entire family. In the meantime his wife had been observing his movements, and had fled to a neighbour's house. He submitted quietly to be taken to the county receptacle for lunatics. When his wife visited him, several days afterwards, he made a violent effort to attack her. He refused food, but usually managed to secrete a portion of that brought to him, and partook of it unobserved. For a period of three months after his admission to the Asylum he maintained a uniform silence. He is now improving, and writes affectionately to his family. He has never disclosed the delusions under which he has been labouring.

III. *Miscellaneous cases, illustrating certain points having a bearing upon the subject under consideration.*

CASE I.—S., admitted August, 1854. A farmer, aged 35, married, three children, a member of Baptist church, academic education, of good habits, in good circumstances, an honest, industrious man, exceedingly kind in his family, and generally respected. His maternal grandmother and grand-aunt and two maternal uncles were insane. His great-grandfather, grandmother, and an uncle committed suicide, and another uncle attempted it. This man has always had a rather delicate constitution, though he has rarely been sick beyond an occasional attack of dyspepsia. Is generally quiet and reserved, though not unsocial in his habits. Previous to admission, laboured beyond his strength, and began to feel depressed. Thought his family would come to want, and that everything was going to destruction. Still, however, attended to his business affairs. Four weeks before admission he suddenly left his house. His wife followed and he returned. Four days afterward he was missed, and, after several hours' search, was found in a wood, near the house, lying upon the ground, and insensible from loss of blood. He had made several incisions in his neck, and opened a vein in his arm. When revived, he would give no account of himself, but soon afterward told his wife, while watching him at night, to send him to the Asylum, or he might do her some injury. He insisted upon this, and was brought to the institution.

At this period he discovered no delusions, but at times was possessed with a strong desire to kill his family and himself. He felt the approach of these paroxysms, and informed those about him of the fact. At these times he was usually sedate and secretive, with flushed face, eyes sparkling, pleasant and bland in manner, bowels constipated, pulse somewhat accelerated, skin natural, urine scanty, appetite good.

These paroxysms usually continued three or four days. At first they occurred at intervals of two or three weeks, and as he improved in general health became less frequent, and in five months disappeared. He remained in the institution ten months, and left in excellent health of mind as well as body.

After recovery the man gave the following account of himself. For several weeks preceding the attempt at self-destruction he felt oppressed, had pain in his head, and at times confusion of thought. Things about him began to appear gloomy, and at length he became very melancholy. He remembered the conduct of his ancestors, and determined not to entertain the idea of the act which had terminated their existence. He had never spoken to his wife of his hereditary tendency to insanity, but had thought of this in the education of his children. One day, while walking with his children in the orchard, he suddenly had a sense of faintness and giddiness, and sat down. He also felt a throbbing in his head. At the time he had no thought of suicide, but determined to go into the house and tell his wife of his situation and feelings. He did not find her, and while alone it occurred to him that he ought to kill himself. Going where his razors were kept, he took one, but immediately put it back, determined to resist, and ran out of the house to the place where he was found. There he was so overcome with the thought that it was his duty to kill himself that he yielded. After his recovery from the effects of this attempt, the impression that he ought to take the lives of his wife and children and himself became at times so strong that he feared he should do it, and requested to be taken to the Asylum.

After several months his wife visited him, and was anxious to take him home. He was, however, unwilling to go, as he did not feel entirely natural at times, and feared that his return might again develop the dreadful propensity. At the end of ten months from his admission his wife again visited him, and after remaining in the city three days, he seeing her daily, and experiencing no unnatural feelings toward her, he returned home, where he has since continued well and happy.

Case II.—S., admitted July, 1856. Aged 51, married, a physician, member of Baptist church, and of good moral habits. Mother was hysterical. Sister has long been and is now insane. When young was addicted to self-abuse. Always somewhat hypochondriacal. In 1849 had an attack of cholera, which left him with permanently impaired health. At four different times, while suffering from indigestion, felt impelled to kill his children. To relieve his sufferings he was accustomed to abstain from food, and on some occasions fasted from one to sixteen days, taking only a little at long intervals. At such times he was in great mental agony.

About seven months before admission, and during a period of abstinence, he was retrospecting his life, and recalled the following trifling incident. In youth he once, with others, in jest, threw an apple paring over his head, which in falling was, by popular impression, supposed to form the letter of the alphabet commencing the name of his future wife. He now remembered that this letter was not the initial of his wife, and therefore he had married the wrong person. He immediately resolved to kill his wife, and the impression increased that it was his duty; "something seemed to say, Kill her, kill her," and he began to fear he should. He appreciated the nature of the crime, and felt that it was in opposition to all the convictions of his life; yet it seemed his duty. In great distress he communicated his fears to his family, and eventually left home and remained away five months. After his return the same unhappy impressions came upon him with increased force. He feared he should destroy his whole family, and requested his son to chain him at night. His son complied with his request, and he felt more comfortable. In a few days he urged his son to keep him constantly chained, or he would certainly kill his family, and then destroy himself. He considered himself a lost man, and given over to the worst passions. For ten days before reception was constantly chained, except while taking his meals, and seemed at times in great agony when considering his condition.

One afternoon, while walking through the room, told his son his conscience was gone, and he would now kill his wife and daughter; but refused to disclose the proposed means. The same afternoon made an unsuccessful attempt to cut his throat. Paroxysms of excitement occurred at irregular intervals.

His domestic relations had always been pleasant, and he still has the most kindly feelings towards his family. When asked if he did not desire to get well, replied, "If I should get well, it would only be on the condition that I then return and murder my family, and cut my own throat. Now I dare not kill myself, because I am commanded first to kill my family." He complains of various peculiar sensations—as though the back of his head was pressed, and of pain running up his back and into the base of the brain, and then spreading out in every direction, sometimes as though fire was flashing through his brain. Form of disease, dementia, which increases. Still retains the idea that he must kill his family,

CASE III.—L., admitted November, 1855. Male, aged 58, married, a farmer, well educated, member of church, and of good moral habits. Received an injury to the spine, which affected his general health. Began to entertain suspicions of those about him. Thought that slanders calculated to injure him were being circulated; that people pursued him, and often declared he heard voices near him. Became much excited, and secured his house against robberies, which he constantly anticipated would be undertaken. These unhappy feelings continued until he at length contemplated suicide as an escape. It then occurred to him that if he did this his wife would be left to cruelty and outrage. He therefore determined to kill her first. This idea became so pressing that, fearing he could not resist it, he communicated his apprehensions to a labouring man, and employed him to sleep in a room communicating with his own, instructing him to enter if he should hear any struggle, and prevent the commission of crime. This watchfulness over himself he continued for a long time, against the remonstrances of his wife, who knew nothing of his murderous feelings, and to whom he declined giving reasons for his conduct. He requested to be brought to the asylum, where he subsequently declared the reasons for this and other acts incomprehensible to his friends.

While in the institution he manifested at times extreme mental distress, accompanied by a disposition to homicide, as well as suicide. The former was incited by his delusions, which were intimately associated with those about him. During his treatment, for a period of five months, the violence of his disease gradually abated, and he returned to his family, resuming the entire management of his affairs, which he continues to the present time.

### ANALYSIS OF FIFTY-TWO CASES OF INSANITY MARKED BY A DISPOSITION TO HOMICIDE.

*Sex.*—Of those who committed the act, nineteen were males and five females; of those who made unsuccessful attempts, twenty were males and five females.

*Habits.*—Of the entire number (fifty-two), twenty-three were intemperate, or vicious, bad men, and twenty-nine were of unexceptionable character and habits.

*Hereditary Predisposition.*—In twenty-one of the fifty-two cases there existed a marked hereditary predisposition, in nine no such predisposition existed, and in twenty-two no facts touching this point were ascertained.

*Mental Disease.*—The form of mental disease was acute mania in fourteen cases, sub-acute mania in three, paroxysmal mania in two, chronic mania in four, dementia in twenty-four, melancholia in four, mania â potu in one. Four of the cases of mania and one of dementia were accompanied by epilepsy.

*Time.*—Twenty-two of the twenty-four homicides were committed in the daytime, the remaining two in the early part of the evening. Of the twenty-five attempts, twenty-one were made in the daytime, two in the night, and two both in the day and night.

*Object of Attack.*—A father was the victim in one case, a brother-in-law in one, a husband in one, wives in four, children in ten, a cousin in one, neighbours in four, neighbours' children in three, and entire strangers in seven cases. In nearly the same proportion, the immediate relations of the patients were the objects of attack in those cases in which the attempt was unsuccessful.

*Suicidal Disposition.*—In ten of the fifty-two cases a suicidal tendency accompanied the disposition to homicide.

*Commitment to Asylum.*—Of the twenty-four homicides, eleven were acquitted by the courts before which they were arraigned, on ground of insanity, and ordered to the asylum; one was found guilty, but sentence was suspended; four were sent here on preliminary trial; six without any criminal proceedings; and two were placed in the asylum by their friends.

*Results.*—Of the twenty-four patients who committed homicide, seven recovered, eleven are unimproved, two eloped, and four have died. Of the twenty-five patients who were prevented from carrying their homicidal purpose into execution, eight recovered, thirteen are unimproved, and four have died.

Arranging these cases under Dr. Bucknill's very convenient modified classification of Esquirol,* we have the following result:—

1. "Those wherein the crime has been occasioned by delusion, and no reasonable person can doubt or object to the irresponsibility of the offender." In this class we have thirty-four of the fifty-two cases.

2. "Wherein the offender, though suffering from cerebro-mental disease, has committed the crime under the influence of some motive not of a delusive character." In this class we have seven of the fifty-two cases.

3. "Where with general symptoms of cerebro-mental disease neither delusion nor motive for the crime are discernible." In this class we have eleven of the fifty-two cases.

Homicidal monomania is one of those unhappy terms in medicine which, while it aims to express the character of the peculiar cases intended to be included under it, is rather the expression of a theory never yet fully established. Esquirol, in his classification, regards it as a partial alienation, and divides the cases generally into homicides committed or contemplated under insane convictions, the monomaniac being influenced by an irrational motive, and always presenting unequivocal indications of a partial derangement of the understanding and feelings; and homicides committed or attempted under a blind, irresistible impulse to the commission of murder, the monomaniac displaying no perceptible disturbance of the understanding or feelings, and his motives not acknowledged or discoverable.

Pinel says, "There are madmen in whom there is no perceptible aberration of the intellectual processes, of the perceptions, judging faculty, imagination or memory, and yet a perversion of the manifestations of the will in a blind impulse to the commission of violence and bloodthirsty rage, without any assignable dominant idea, or any delusion of the imagination which could cause such a propensity."

The opinions of these distinguished authorities on this subject have been entertained and reiterated by subsequent writers down to the present time, although the existence of the impulsive variety, not fully established even in its day, has been more and more doubted, until it has enrolled against it many of the most able writers on the jurisprudence of insanity in Europe and America. Among those who either reject or question the whole theory of moral insanity, under which this monomania is placed, are found the names of Heinrich, Leubuscher, Winslow, Mayo, Bucknill, and Wharton.

* Bucknill on Criminal Lunacy, page 100.

That in insanity there is developed a disposition to the extreme violence of murder, and that this disposition is at times irresistible, no one familiar with the insane will for a moment pretend to doubt; and from such observation as cases have thus far afforded, we cannot but adopt the opinion that the morbid tendency to homicide is but one of the many violent impulses of the insane state—one among the many manifestations of perverted instinct exhibited *in* the disease; and that the homicidal act, in irresponsible persons, generally, if not always, has for its origin and development such motives or disturbance of feelings as usually influence the insane to other carefully planned or sudden acts of violence; and that a full and reliable history of cases of sudden or more or less persistent homicidal propensity will reveal the fact, that, *in all instances,* anterior to any such impulse, there existed for a time *physical disease,* or at least perceptible disturbance of the physical health and a change in the mental condition; or, in other words, *a state of insanity.*

In noticing Esquirol's classification of homicidal insanity, Dr. Bucknill, editor of the *Asylum Journal of Mental Science,* one of the ablest modern writers, and with a large practical experience of the insane, remarks:—

"The existence of the third class, in which the impulse is sudden and unreflected on, admits of grave doubt. The testimony in favour of the existence of such a variety is very scanty and unsatisfactory: and it is improbable that cerebro-mental disease can develop itself in so rapid a manner. It is probable that the cases of insanity which have been placed under this head were less recent and sudden than they were supposed to be. The earlier stages of diseased feeling had been unobserved by others, and unacknowledged by the patient."*

On the use of the term "impulse," the same author says:— "It conveys the idea of force communicated instantaneously, a rapid motive; whereas the morbid desires under consideration are not of instantaneous production, or of rapid growth. They arise from a chronic disease, and are resisted up to a certain point; sometimes they are altogether and successfully resisted; sometimes, unhappily, they prove too strong for the power of the will. In order to establish this form of insanity, the existence of a diseased emotion must be proved. The will itself is a faculty so simple and undecomposable that it may be doubted whether it can ever lapse into a diseased condition."

Mr. Wharton, one of the ablest American writers, in his recent valuable treatise on Medical Jurisprudence, uses the following language:—

"The term monomania, however, is only admissible in so far

* Bucknill on Criminal Lunacy, page 83.

as it designates certain fixed objects, towards which the ravings of the maniac are directed, and which supply the apparent motives of his actions; *it is not to be supposed that a single impulse is diseased, while all the other functions of the mind retain their healthy action.* While the entire intellect enjoys sound health, there is nothing in which a morbid desire of theft, murder, &c., could originate; and such a phenomenon is a psychological impossibility, and the assumption of such requires a psychological contradiction. A mania without delirium, a mania without a morbid participation or disturbance of the perceptive faculties, is, therefore, out of the question; as a desire to injure or destroy is impossible without an act of the mind by which this purpose is entertained, and as reason and understanding are alike disordered, whether they insinuate a wrong motive for the morbidly conceived purpose of the act, or whether they entirely omit the suggestion of any reason whatever." Again: "Where there is no will, but only a blind impulse, *a perversion of the manifestations of the will is not to be supposed.*"*

Esquirol's class, then (commonly designated as "impulsive homicidal mania"), in consequence of its tendency to embrace within his definition of it, and therefore to shield from punishment, a great mass of crimes of the most atrocious and appalling character, should, notwithstanding the justly exalted reputation of its author, be exposed to the test of the most convincing facts and experience before it is allowed to stand, especially in the criminal tribunals, as an authorized and distinctive classification of insanity. That such a form of insanity has been enunciated by Esquirol, or by other acknowledged authorities in the profession, is not in itself an evidence of its real existence. Errors, in medical science as well as in other sciences, creep on from generation to generation, and even become fortified by time and tradition, until they are finally exposed by a series of facts and observations, carefully analyzed and collated, which fix the truth beyond any reasonable cavil.

Violence against the person and against property is now so prevalent, that we should be extremely cautious of recognising any doubtful form of insanity that will ever shield it; and unless we know, by accurate inquiry and observation, that some form or other of physical disease, remote or direct, has produced a *state of insanity*, it is not just to society to claim for any homicide, or other act of violence or wrong, the protection of such a defence. The closer accuracy of investigation into the history of cases which is now demanded, and which is bestowed by a multitude of observers, certainly shows, on a careful analysis of its results,

* Wharton and Stillé's Medical Jurisprudence, page 145.

that many cases which might heretofore have been classed as "impulsive homicide" do not justify the epithet of "impulsive." Some are *homicidal* in the guilty sense only, and others are *homicidal* as a result of insanity in its proper and common meaning, as understood by the law and conceded by the popular sentiment. More accurate information and more thorough observation might, and probably would, have detected, in most cases intended by Esquirol to be comprehended in his "*impulsive*" class, such a state of present disease, or of long-standing insane tendencies, as would, in his own mind, have resolved this class into some other of distinct and well-acknowledged insanity, not at all (in his sense of the term) impulsive; or discovered such a state of evil habitudes as would have thrown them into the class of criminals deserving of no particular clemency.

The cases we have submitted seem to favour these suggestions and conclusions; and the facts, we trust, will aid in shedding a more certain light upon a subject often obscure and perplexing, and confessedly of the highest moment to individuals as well as to society.

---

